# Addressing ethnic disparities in neurological research in the United Kingdom: An example from the prospective multicentre COVID-19 Clinical Neuroscience Study

**DOI:** 10.1016/j.clinme.2024.100209

**Published:** 2024-04-19

**Authors:** Daniel J. van Wamelen, Silvia Rota, Monika Hartmann, Naomi H. Martin, Ali M. Alam, Rhys H. Thomas, Katherine C. Dodd, Thomas Jenkins, Craig J. Smith, Michael S. Zandi, Ava Easton, Georgina Carr, Laura A. Benjamin, James B. Lilleker, David Saucer, Alasdair J. Coles, Nicholas Wood, K. Ray Chaudhuri, Gerome Breen, Benedict Daniel Michael, COVID-CNS consortium

**Affiliations:** a Institute of Psychiatry, Psychology & Neuroscience; Department of Neuroimaging; King's College London, London, United Kingdom; b Parkinson Foundation Centre of Excellence at King's College Hospital NHS Foundation Trust, and King's College London, United Kingdom; c Radboud University Medical Center, Donders Institute for Brain, Cognition and Behaviour, Department of Neurology; Centre of Expertise for Parkinson & Movement Disorders, Nijmegen, the Netherlands; d Institute of Psychiatry, Psychology & Neuroscience, Department of Basic & Clinical Neuroscience, Division of Neuroscience; King's College London, London, United Kingdom; e Institute of Infection, Veterinary and Ecological Science, University of Liverpool, NIHR HPRU for Emerging and Zoonotic Infection, Liverpool, United Kingdom; f Translational and Clinical Research Institute, Newcastle University, Newcastle upon Tyne, United Kingdom; g Royal Victoria Infirmary, Queen Victoria Road, Newcastle upon Tyne, United Kingdom; h Lydia Becker Institute of Immunology and Inflammation, Faculty of Biology, Medicine and Health, University of Manchester, Manchester Academic Health Science Centre, Manchester, United Kingdom; i Manchester Centre for Clinical Neurosciences, Salford Royal Care Organisation, Northern Care Alliance NHS Foundation Trust, Salford, United Kingdom; j Department of Neurology, Sheffield Teaching Hospitals NHS Foundation Trust, Sheffield, United Kingdom; k Sheffield Institute for Translational Neuroscience, University of Sheffield, Sheffield, United Kingdom; l Midland St John of God Hospital and Curtin University, Perth, Western Australia, Australia; m Division of Cardiovascular Sciences, School of Medical Sciences, The University of Manchester, Geoffrey Jefferson Brain Research Centre, Manchester, United Kingdom; n National Hospital for Neurology and Neurosurgery, London, United Kingdom; o Encephalitis Society, Malton, United Kingdom; p Neurological Alliance, London WD17 1EU, United Kingdom; q Stroke Research Centre, UCL Queen Square Institute of Neurology, London, United Kingdom; r Centre for Musculoskeletal Research, Division of Musculoskeletal and Dermatological Sciences, School of Biological Sciences, Faculty of Biology, Medicine and Health, Manchester Academic Health Science Centre, The University of Manchester, Manchester, United Kingdom; s Department of Clinical Neurosciences, University of Cambridge, Cambridge, United Kingdom; t Department of Clinical and Movement Neurosciences, University College London, UCL Queen Square Institute of Neurology, London, United Kingdom; u Institute of Psychiatry, Psychology & Neuroscience; Social, Genetic & Developmental Psychiatry Centre; King's College London, London, United Kingdom; v The Walton Centre NHS Foundation Trust, Department of Neurology, Liverpool, United Kingdom

**Keywords:** Ethnicity, Diversity, Recruitment, Neurology, COVID-19

## Abstract

**Background:**

Minority ethnic groups have often been underrepresented in research, posing a problem in relation to external validity and extrapolation of findings. Here, we aimed to assess recruitment and retainment strategies in a large observational study assessing neurological complications following SARS-CoV-2 infection.

**Methods:**

Participants were recruited following confirmed infection with SARS-CoV-2 and hospitalisation. Self-reported ethnicity was recorded alongside other demographic data to identify potential barriers to recruitment.

**Results:**

807 participants were recruited to COVID-CNS, and ethnicity data were available for 93.2%. We identified a proportionate representation of self-reported ethnicity categories, and distribution of broad ethnicity categories mirrored individual centres’ catchment areas. White ethnicity within individual centres ranged between 44.5% and 89.1%, with highest percentage of participants with non-White ethnicity in London-based centres. Examples are provided how to reach potentially underrepresented minority ethnic groups.

**Conclusions:**

Recruitment barriers in relation to potentially underrepresented ethnic groups may be overcome with strategies identified here.

## Introduction

Increasing the diversity of study participants in order to represent the general population, is of crucial importance to allow research findings to be translatable, and to enable the personalisation of treatment and care. Addressing ethnicity-related inequalities in research participation is of particular importance in countries or regions with a multi-ethnic population. Inclusion of underrepresented groups is imperative to determine not only possible differences in disease outcomes, but also treatment response among ethnic groups – as shown in some neurological conditions, such as Parkinson's disease.^1^ Currently, many neurological studies fail to address the diversity of participants in research, despite historical patterns of underrepresentation for minority ethnic groups, women and older adults being well recognised.^2^ For example, a recent meta-analysis demonstrated the underrepresentation in stroke studies in North America, showing that non-White participants comprised only 23% of participants in studies, despite an increased risk of stroke and stroke recurrence in some non-White groups, such as African Americans.^3^, ^4^, ^5^ It is not fully understood why under-representation of minority ethnic groups occurs, although in some interventional studies, such as for vascular neurology, this may sometimes differ between acute and chronic interventions.^6^^,^^7^ In addition, even in observational studies, potential barriers for some may include mistrust in medical research arising from historical abuse and contemporary biased systems, cultural and language barriers, low socioeconomic status, and lack of awareness of research studies.^8^, ^9^, ^10^ The consequences of the pandemic may have exacerbated existing health disparities. The mortality and morbidity burden of acute COVID-19 infection was disproportionately felt by minority ethnic groups and communities, who were also less likely to receive telehealth services.^11^

Here, we aimed to assess recruitment and retainment strategies in a large observational study prospectively recruiting hospitalised patients with neurological complications following SARS-CoV-2 infection and a control group of hospitalised patients with COVID-19, but without neurological complications. In this post-hoc analysis, we sought to evaluate ethnic diversity by geographical region, to identify potential barriers to recruitment and retention, and to provide examples of strategies to increase ethnic diversity across study populations, which has implications for neurological studies in general.^12^^,^^13^

## Methods

Data described in this manuscript were obtained from the COVID-19 Clinical Neuroscience Study (COVID-CNS; www.covidcns.org), a multi-centre observational study in the UK, including 17 centres across England and Wales, addressing the need to understand the clinico-epidemiologic spectrum and biological causes of neurological and neuropsychiatric complications in hospitalised patients with COVID-19, caused by an infection with Severe Acute Respiratory Syndrome Coronavirus-2 (SARS-CoV-2). This study was added as a separate cohort study embedded within the existing NIHR BioResource - Research Tissue Bank study and obtained ethical approval in the UK (REC 17/EE/0025, IRAS 220277). All participants gave written informed consent, and all procedures were performed in accordance with the declaration of Helsinki.

We obtained data regarding age, sex, level of education, relationship status, and self-reported ethnicity, collected as part of the study protocol. The latter data consisted of broad ethnicity groups as derived from NHS guidance used in the United Kingdom^14^: i) Asian, ii) Black, iii) Mixed, iv) Other, and v) White. Demographic information was reported using the recently published Updated Guidance on the Reporting of Race and Ethnicity in Medical and Science Journals.^13^ Furthermore, we included admission related data, including days spent in hospital, World Health Organisation (WHO) severity scores for COVID-19,^15^ and duration of invasive ventilation.

Information regarding the distribution about the UK population in terms of broad ethnicity categories was collected from the Office for National Statistics website.^14^ We examined ethnicity distribution only for centres that recruited at least 50 participants to the COVID-CNS studies; centres excluded for the current analyses had a median of eight participants (range 1–30). For centres included in the current analysis, their respective Clinical Catchment Area was determined as follows, based on the National Health System's organisational oversight,^16^ described as (centre (local authority)): the Walton Centre and Liverpool University NHS Foundation Trust (Liverpool City Region), Salford Royal (Greater Manchester), Cambridge University Hospitals (Cambridge), Sheffield Teaching Hospitals (Sheffield), University College Hospital (London Boroughs of Camden, Islington, Haringey, Barnet, and Enfield), and King's College Hospital (London Boroughs of Bexleyheath, Bromley, Greenwich, Lambeth, Lewisham, and Southwark). For each catchment area the distribution of broad ethnicity categories was obtained for each borough with the above defined catchment from the Office of National Statistics website with data obtained from the most recent census in the UK (Census 2021).^14^^,^^17^ A weighted average for each broad ethnicity category was calculated for each catchment area using the UK Census 2021 for ethnicity and population.^14^^,^^17^

Data were summarised descriptively and where data were normally distributed, they are presented as mean ± standard deviation and analysed using student *t* test. For non-parametric data, data are represented as median (range) and analysed by Mann–Whitney *U* or Fisher's Exact test. For dichotomous group comparisons, a Chi-Square test was used. A correction for multiple testing, where relevant, was performed using the Bonferroni method. All data were analysed using SPSS Version 28 (IBM SPSS Statistics for Windows, Version 28.0. Armonk, NY: IBM Corp.).

## Results

**Fig. 1:**
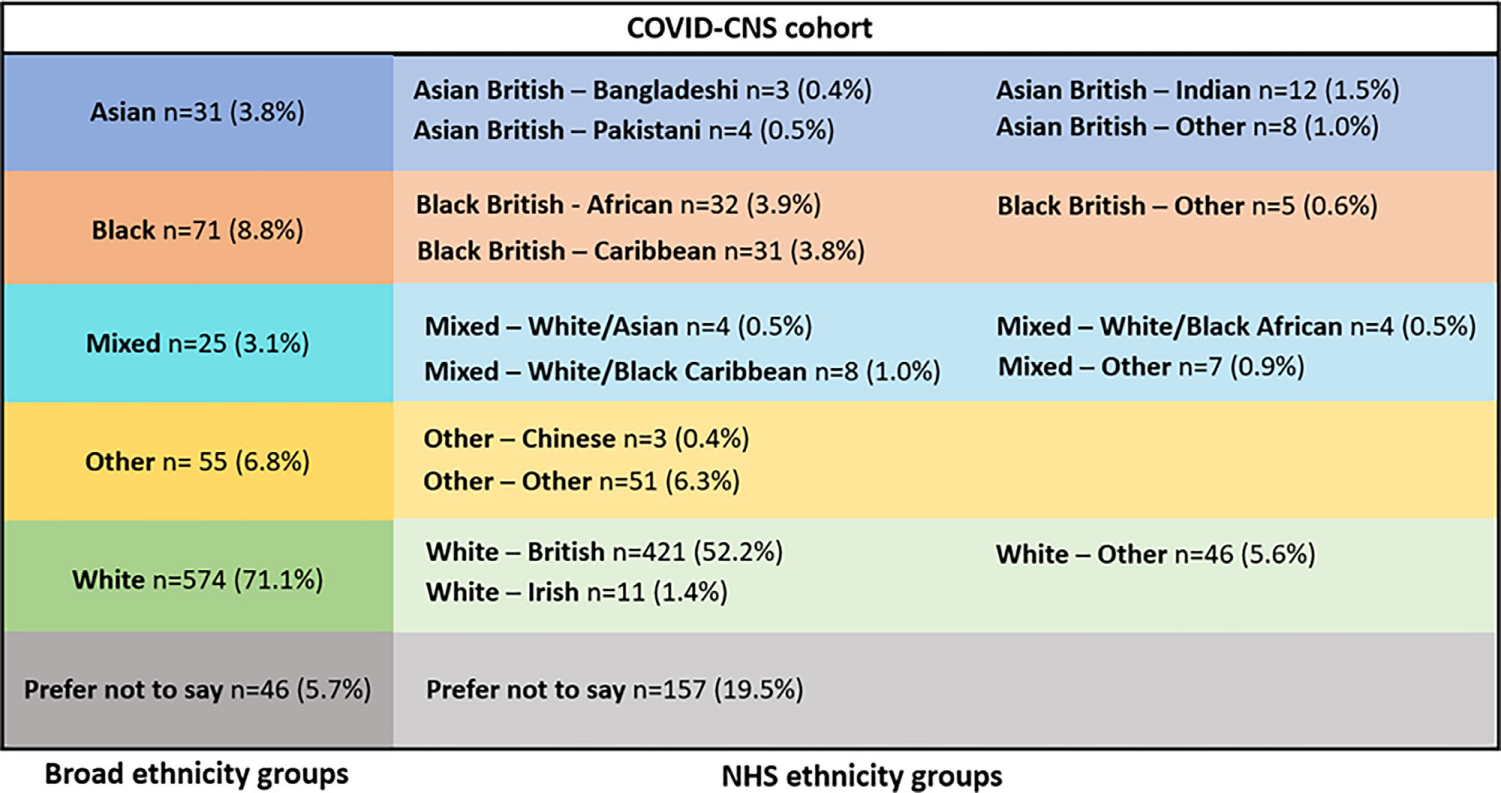
Ethnicity in the COVID-CNS study. Please note that the numbers and percentages for broad ethnicity and NHS ethnicity groups do not necessarily match due to differences in the 'Prefer not to say' items. Abbreviations: NHS: National Health Service.

**Fig. 2:**
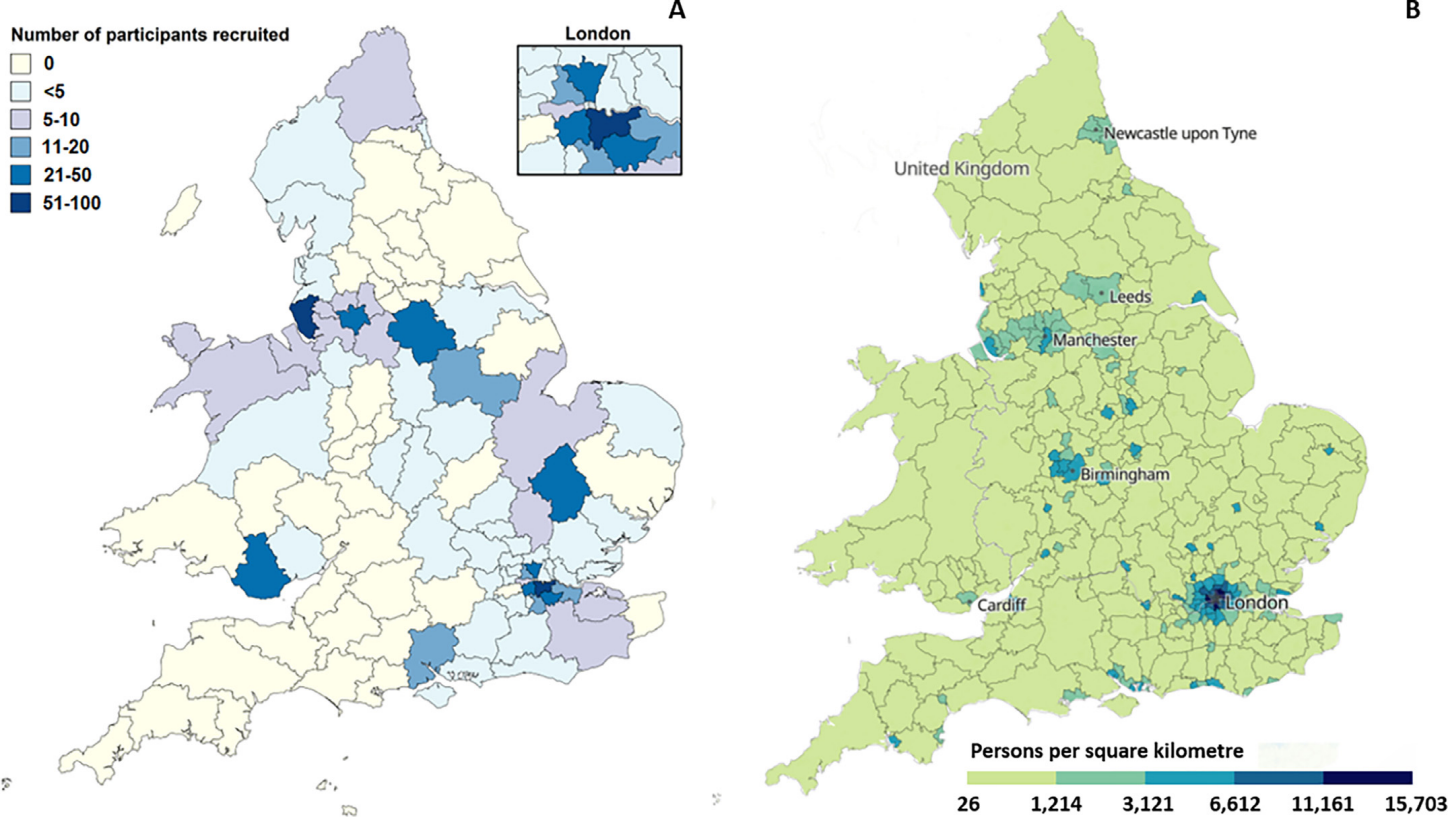
Geographical distribution of participant recruitment for the COVID-CNS study relative to overall population. Fig. 2A shows England and Wales with the number of participants in the COVID-CNS study per postcode area; Fig. 2B shows overall population density across England and Wales (created using Census 2021 data from the Office for National Statistics; https://www.ons.gov.uk/census/maps).

Baseline demographics for the participants in the COVID-CNS study are provided in Table 1, an overview of ethnicity in Fig. 1, and the geographical distribution of participant recruitment relative to overall population density is shown in Fig. 2. We identified variety in the distribution of ethnic minority participation across centres that participated in the COVID-CNS consortium in terms of broad ethnicity groups (Table 2), but for the overall COVID-CNS cohort, as well as the individual included centres, the distribution of broad ethnicity groups was comparable to their Clinical Catchment Areas (Table 3). Moreover, when comparing non-White versus White participant percentages, no differences were observed in the distribution between the centres Clinical Catchment Areas and the Census 2021 data, showing that the COVID-CNS study was able to recruit a cohort of participants representative of the UK population in terms of ethnicity (Table 3). In addition, there was a generally good representation of minority ethnic groups and no differences in distribution of ethnicity groups was observed between male and female participants (*p* = 0.507). In addition, female participants more often tended to be from an ethnic minority group compared to male participants (31.2% of female participants were from a non-White ethnic background, whereas this was 26.6% for male participants), although this did not reach statistical significance (*p* = 0.113).

**Table 1:** Baseline demographics.

**Age (years)**	54.58±14.96
**Sex (M/F)**	459/343 (56.9%/42.5%)
**Relationship status** *Single* *Partner* *Married* *Divorced* *Widowed* *Other or prefer not to say*	102 (12.6%) 97 (12.0%) 354 (43.9%) 39 (6.1%) 28 (3.5%) 177 (21.9%)
**Highest education** *A levels or equivalent* *O levels or equivalent* *College or university degree* *CSEs or equivalent* *NVQ or equivalent* *Other professional degree* *None of the above* *Unanswered or prefer not to say*	53 (6.6%) 132 (16.4%) 252 (31.2%) 40 (5.0%) 46 (5.7%) 22 (2.7%) 79 (9.8%) 184 (22.7%)
**Broad ethnicity groups** *Asian* *Black* *Mixed* *Other* *White* *Prefer not to say*	31 (3.8%) 71 (8.8%) 25 (3.1%) 55 (6.8%) 574 (71.1%) 46 (5.7%)

Abbreviations: M: male; F: female; CSE: certificate of secondary education; NHS: National Health Service; NVQ: national vocational qualification. Note that discrepancies may be present between NHS ethnicity categories and broad ethnicity categories due differences in the ‘prefer not to say’ item for respective categories.

**Table 2:** Ethnicity across the larger recruiting centres participating in the COVID-CNS consortium. Only centres who recruited at least 50 participants have been included in this overview.

Broad ethnicity categories	Asian	Black	Mixed	Other	White	Undeclared	p*
King's College Hospital London (n=227)	10 (4.4%)	50 (22.0%)	9 (4.0%)	29 (12.8%)	101 (44.5%)	27 (11.9%)	N/A
The Walton Centre Liverpool (n=193)	2 (1.0%)	5 (2.6%)	7 (3.6%)	2 (1.0%)	172 (89.1%)	5 (2.6%)	N/A
Cambridge University Hospitals Cambridge (n=78)	1 (1.3%)	2 (2.6%)	0 (0.0%)	1 (1.3%)	62 (79.5%)	12 (15.4%)	N/A
University College Hospital London (n=60)	6 (10.0%)	4 (6.7%)	2 (3.3%)	11 (18.3%)	35 (58.3%)	2 (3.3%)	N/A
Salford Royal Hospital Salford (n=72)	2 (2.8%)	3 (4.2%)	3 (4.2%)	4 (5.6%)	60 (83.3%)	0 (0.0%)	N/A
Sheffield Teaching Hospitals Sheffield (n=71)	3 (4.2%)	1 (1.4%)	2 (2.8%)	3 (4.2%)	60 (84.5%)	2 (2.8%)	N/A

**Broad ethnicity categories**	**Asian**	**Black**	**Mixed**	**Other**	**White**	**Undeclared**	p*

Age (years)	47.3±13.2	56.3±14.6	48.2±13.6	54.2±13.6	54.8±14.7	58.0±18.9	**0.001^a^**
Body weight (kg)	79.2±15.9	88.3±20.3	88.2±23.7	87.7±18.7	88.5±20.0	108.0±35.9	0.117
Duration of invasive ventilation (days)	9.2±19.4	4.5±11.0	4.5±10.2	3.5±8.6	3.5±10.3	0.3±1.7	0.091
Duration of admission (days)	25.2±31.0	20.0±28.4	17.2±23.2	19.1±23.2	22.0±36.4	16.9±27.6	0.599
WHO severity scores	4.4±0.7	4.6±0.9	4.4±1.0	4.5±0.7	4.4±0.9	4.3±0.7	0.172

Abbreviations: NHS: national health service; WHO: World Health Organization; N/A: not applicable; p*: level of statistical significance after Bonferroni correction *p* = 0.01 (0.05/5); a: post-hoc *p* < 0.05 between Asian and Black, Asian and White, and Asian and undeclared.

**Table 3:** Ethnic background in the COVID-CNS study and individual centres compared to ethnic diversity in the United Kingdom and local centre catchment areas. Catchment areas were defined as follows (centre (local authority)): the Walton Centre (Liverpool), Salford Royal Hospital (Greater Manchester), Cambridge University Hospitals (Cambridge), Sheffield Teaching Hospitals (Sheffield), University College Hospital (London Boroughs of Camden, Islington, Haringey, Barnet, and Enfield), and King's College Hospital (London Boroughs of Bexleyheath, Bromley, Greenwich, Lambeth, Lewisham, and Southwark).

COVID-CNS centre	Broad ethnicity category	COVID-CNS	Census 2021 area	*p*-values
Entire cohort	Asian Black Mixed Other White Unknown	3.8% 8.8% 3.1% 6.8% 71.1% 5.7%	9.3% 4.0% 2.9% 2.1% 81.7%	0.994^a^ 0.918^b^
The Walton Centre Liverpool	Asian Black Mixed Other White Unknown	1.0% 2.6% 3.6% 1.0% 89.1% 2.6%	2.9% 3.3% 2.1% 5.3% 92.1%	1.000^a^ 0.984^b^
Salford Royal Hospital Salford	Asian Black Mixed Other White Unknown	2.8% 4.2% 4.2% 5.6% 83.3% 0.0%	12.6% 4.7% 3.0% 3.1% 76.4%	0.999^a^ 0.872^b^
Cambridge University Hospitals Cambridge	Asian Black Mixed Other White Unknown	1.3% 2.6% 0.0% 1.3% 79.5% 15.4%	14.8% 2.4% 5.1% 3.1% 74.5%	0.994^a^ 0.657^b^
Sheffield Teaching Hospitals Sheffield	Asian Black Mixed Other White Unknown	4.2% 1.4% 2.8% 4.2% 84.5% 2.8%	9.6% 4.6% 3.5% 3.2% 79.1%	1.000^a^ 0.846^b^
University College Hospital London	Asian Black Mixed Other White Unknown	10.0% 6.7% 3.3% 18.3% 58.3% 3.3%	13.9% 13.1% 6.3% 9.5% 57.2%	0.997^a^ 0.950^b^
King's College Hospital London	Asian Black Mixed Other White Unknown	4.4% 22.0% 4.0% 12.8% 44.5% 11.9%	9.5% 19.5% 6.5% 4.3% 60.2%	0.992^a^ 0.847^b^

Abbreviations: a: across all groups; b: non-white vs white.

To determine if any bias was present relating to inclusion of participants from different ethnic backgrounds, we next determined if demographic and COVID-19 related differences existed between different broad ethnicity groups. After correction for multiple testing through the Bonferroni method (*p* = 0.05/6 = 0.008), no statistically significant differences were observed for sex, body weight, the number of days spent in hospital, WHO severity scores, duration of invasive ventilation (Table 2) between broad ethnicity category groups, but we did observe a statistically significant difference in age, with participants from Asian and Mixed ethnic minority groups tending to be younger than participants from other groups (*p* = 0.001; Table 2).

Finally, to identify factors that contributed to the comparable distribution of broad ethnicity groups between the COVID-CNS study and the Clinical Catchment Areas of participating centres, researchers working on the study and our PPIE group were retrospectively asked to identify strategies they used to increase participant diversity. The identified strategies are listed in Table 4.

**Table 4:** Strategies used in the COVID-CNS study to increase participant diversity.

Culturally sensitive and diverse research team	• Having a diverse research team from different ethnic backgrounds • Ensuring that the research team is culturally sensitive and motivated to support diverse participation • Video testimonials from participants acting as patient ambassadors on study website
Overcoming financial/social barriers	• Reimbursement of travel expenses / arranging travel for participants unable to pay in advance • Support completing the online follow-up questionnaires over the phone for participants without internet access • Evening sessions to fit around work schedules • Session times to fit around childcare needs
Overcoming language and communication barriers	• Aural consent form for visually impaired participants • Accessing translation services for those for whom English was not a first language • Patient testimonial videos to ensure people from ethnic minority groups could identify with participants already recruited to the study
Meeting participant's individual needs	• Allowing relatives to attend appointments • Working alongside caregivers to ensure both parties’ needs are met • Giving anxious participants alternative ways to provide biosamples • Shorter and fractioned sessions • Providing a calm and suitable environment for participants

## Discussion

Here we provide an example of the successful recruitment of individuals from ethnic minority backgrounds in the UK to the COVID-CNS study. We observed a high degree of diversity in ethnic background and demonstrated which strategies may have helped to achieve this level of diversity by focusing on the recruitment approaches. While acknowledging the limitations of a largely *post hoc* approach in analysing these factors, we feel that with the use of these strategies, a similar degree of diversity in ethnic background in research studies can be observed as in the general population. The strategies employed by the COVID-CNS research teams may prove helpful in other neurological studies and trials and tie in with the advice of widening participation of underrepresented groups in research which is major agenda item for funders eg the NIHR.^18^

When asked about barriers to recruitment of minority ethnic groups, the replies given by the COVID-CNS research teams were largely in line with known barriers described in literature.

Solutions to these identified barriers are crucial and seem to be dependent on the background of specific minority ethnic groups. Although most of the evidence seems to stem from non-neurological studies, much can be learned from advancements in other fields. For example, in a study on hypertension self-management, core factors that aided in the recruitment in an African American population included the presence of a culturally sensitive and diverse research team, in addition to the use of incentives.^19^ Specifically, the study team consisted of individuals from a diverse ethnic background, and the study used monetary incentives to increase retainment. Other factors that contributed to the high recruitment numbers (96.7% over a period of 7 months) and low attrition (16.9% after 6 months), included having previous experience with the study population, working closely with other staff at the study site, and ongoing communication with all parties involved in the study.^19^ This has also been shown in other studies where individuals from underrepresented racial and ethnic groups feel more confident about participating when the research team approaching them is led by people from the same ethnic background.^20^ These strategies are largely in line with recently described approaches for neurological studies. This includes the 'King's Model for Minority Ethnic Research Participant Recruitment', which raises awareness about and supports the recruitment of minority ethnic groups in neurological and other studies and underlines the key unmet need of validating clinical research outcomes in non-white populations.^21^

Another key factor to consider is addressing language and communication barriers. This is exemplified by an over 80% satisfaction among Hispanic American participants in relation to a Spanish translation of clinical information in the same format as the English information provided for a study.^22^ In COVID-CNS, translation services for study documents and research appointments were available. It is important to ensure studies are appropriately funded to cover translation requirements. Other examples of successful recruitment, relevant to COVID-19 studies, include the performance of different COVID-19 vaccine studies. For example, the Novavax study recruited the highest proportion of individuals from a minority ethnic background, which was attributed to the later start of recruitment for the study, enabling a benefit from the ongoing efforts to increase diversity in COVID-19 vaccine trials in general. This also emphasised the need for ongoing engagement and extended recruitment periods as individuals from ethnic minority backgrounds tended to enrol later in the recruitment process, due to strengthened community engagement efforts, and access to more diverse volunteer registry records.^23^ This trend was not observed in the COVID-CNS study and here recruitment of ethnic minority groups occurred evenly throughout the recruitment period.

In addition to a focus on increasing diversity in study populations from a perspective of ethnic background, other issues need to be addressed when it comes to ensuring representative recruitment for clinical trials and studies. For example, those from a low socioeconomic status remain less represented in study recruitment. Addressing this inequality may be achieved by approaching individuals over the phone through a toll-free (freephone) number,^24^ as well as using partners of participants for support, and societal partnership, which have been shown to be effective and can lead to increased enrolment rates.^25^^,^^26^ Moreover, higher retention rates have been observed through use of (financial) incentives, a personalised approach, using project logos, emphasising participant convenience, and sustained contact with participants.^26^ Such strategies may also be of importance when trying to recruit participants from a rural setting where underrepresented minority groups may be more difficult to reach due to geographical isolation and lower population density. Here, the use of multiple recruitment strategies could be particularly beneficial.^27^

Finally, addressing physician referrals is crucial; for example in oncology trials 77% of trial participants reported that it was their physician who made them aware about specific studies.^28^ Surveys show that many physicians do not refer patients to studies due to a lack of time or knowledge about ongoing trials and studies.^29^ Physicians may have unintentional bias to recruiting participants from non-minority backgrounds.^30^ Therefore, engaging with physicians, and aiming to increase involvement of physician from ethnic minority groups, as well as providing suitable information materials about studies could aid in increasing recruitment, particularly among individuals from an ethnic minority background.

As with any study it is important to reflect on limitations of our analyses. These include the possible bias introduced by the greater ethnic diversity in London compared to other parts of the UK. As such, it could be reasoned that the greater ethnic diversity observed in the London centres participating in the COVID-19 study could be explained by greater ethnic diversity in London. On the other hand, we noted that the distribution regarding broad ethnicity categories across the United Kingdom was in line with the Census 2021 data. Moreover, other studies undertaken in the same population in London have not reached the diversity in ethnic background observed in the COVID-CNS study.^31^^,^^32^ It could be argued that, as the nodal event was admission to hospital and people from ethnic minority group were more likely to have higher COVID-19 disease severity,^33^ it was easier to recruit participants from a wider range of ethnic minority groups, also in the light of difficulties some studies on COVID-19 experienced when trying to recruit outpatients.^34^ In this study we have not been able to include data related to eg free childhood meals and exact postcode of participants, which are also determinants of health. Finally, we observed relatively high rates of participants who did not want to indicate their self-reported ethnicity, in addition to the limited ethnicity group options provided by the NHS. Nonetheless, we feel our results are useful and form a useful source of information regarding recruitment of individuals from an ethnic minority background in multi-ethnic countries and regions, also by providing examples of how to successfully overcome barriers to recruitment. This also applies to studies in general, and our examples align with the priority needs and most successful strategies identified in other neurological research, such as engaging in community outreach to build trust and understanding, tailored explanation of the study based on language and cultural background, providing adequate support in relation to time and resources that participants have to invest in study participation, as well as careful scheduling of study visits.^35^

## Concluding remarks

To conclude, in this study we provided an example of the successful recruitment of individuals from ethnic minority backgrounds in the UK. A high degree of diversity in ethnic background was achieved in recruitment, mirroring the ethnic diversity across the general UK population. We demonstrated which strategies could be used to achieve this level of diversity and how further research into identification of barriers to recruitment and strategies is vital in tackling these barriers across clinical trials and studies to enable the correct extrapolation of research findings to the general population.

**Summary box**

*What is known*: Inclusion of Black and minority ethnic communities in clinical neurological research has often been sub-optimal, which poses a significant problem in relation to the external validity and extrapolation of study findings.

*What is the question*: How did the COVID-CNS study, looking at neurological complications of COVID-19 infection, perform in terms of participant diversity and inclusion?

*What was found*: We identified an overall proportionate representation of all broad self-reported ethnicity categories in the participant cohort in the COVID-CNS study, with the distribution of broad ethnicity categories in individual centres largely mirroring that of their respective catchment areas.

*What is the implication for practice now*: Recruitment barriers in relation to ethnic minority background can be overcome with strategies identified in this study.

Alt-text: Unlabelled box

## Author contributions

DvW, SR, KRC, and BDM were involved in conception of the research project, data analysis, and writing of the manuscript. All authors were involved in data collection and critical review of the manuscript.

## Declaration of competing interest

None.
